# Long COVID in Brain Health Research: A Call to Action

**DOI:** 10.3390/brainsci14060587

**Published:** 2024-06-08

**Authors:** Thorsten Rudroff

**Affiliations:** 1 Department of Health and Human Physiology, University of Iowa, Iowa City, IA 52242, USA; thorsten-rudroff@uiowa.edu; 2 Department of Neurology, University of Iowa Hospitals and Clinics, Iowa City, IA 52242, USA

**Keywords:** long COVID, fatigue, cognitive function, neuroimaging, brain stimulation

## Abstract

The COVID-19 pandemic has brought attention to the long-term consequences of the virus, particularly the persistent symptoms that characterize long COVID. This syndrome, which can last for months after the initial infection, includes a range of neurological and neuropsychiatric manifestations that have significant implications for brain health and dementia research. This review explores the current understanding of long COVID’s cognitive, neurological, and psychiatric symptoms and their potential impact on brain stimulation and neuroimaging studies. It argues that researchers must adapt their study designs and screening processes to account for the confounding effects of long COVID and ensure the accuracy and reliability of their findings. To advance the understanding of this condition and its long-term effects on brain health, the review proposes a series of strategies, including the development of standardized screening tools, the investigation of underlying mechanisms, and the identification of risk factors and protective factors. It also emphasizes the importance of collaborative research efforts and international data sharing platforms in accelerating the pace of discovery and developing targeted interventions for individuals with long COVID. As the prevalence of this condition continues to grow, it is imperative that the neuroscience community comes together to address this challenge and support those affected by long COVID.

## 1. Introduction

The SARS-CoV-2 virus, responsible for the COVID-19 pandemic, has infected millions worldwide, leading to a wide range of acute and chronic health consequences [1]. While much attention has been focused on the acute respiratory symptoms of COVID-19, a growing body of evidence suggests that the virus can also have long-term effects on various organ systems, including the brain [2]. Long COVID, a term used to describe persistent symptoms experienced by individuals who have recovered from acute COVID-19 illness, has emerged as a significant public health concern [3]. As brain health research continues, it is essential to consider the potential impact of a history of COVID-19 infection and long COVID symptoms on study outcomes. These symptoms can include cognitive impairment, fatigue, mood disturbances, and other neurological issues that may persist for weeks or months after the initial infection [3].

The prevalence of long COVID varies across studies due to differences in definitions, study populations, and methodologies. A community-based study in England involving 606,434 participants found that the prevalence of persistent COVID-19 symptoms lasting 12 weeks or more was 1.7% overall, with a higher prevalence among women, older individuals, and those with pre-existing health conditions [4]. A systematic review and meta-analysis of 81 studies, including a total of 41,879 COVID-19 survivors, reported a pooled prevalence of fatigue of 32% and a pooled prevalence of cognitive impairment of 22% among COVID-19 survivors in the post-acute phase [5]. Another study analyzing electronic health records of 273,618 COVID-19 survivors found that the incidence of neurological or psychiatric diagnoses at 6 months was 33.6%, with 12.8% receiving such a diagnosis for the first time [6]. These studies suggest that the prevalence of long COVID is significant, with estimates ranging from 1.7% to 76% depending on the study population, duration of follow-up, and specific symptoms assessed [4,5,6,7,8].

Brain health studies, conducted during and after the COVID-19 pandemic, have often overlooked the potential impact of long COVID symptoms on their participants and findings. This oversight may lead to confounding effects and inaccurate conclusions, as long COVID has been associated with a range of neurological and neuropsychiatric manifestations that can significantly influence brain health outcomes [1].

## 2. The Impact of Long COVID on Brain Health and Dementia

A recent review article [9] examined the effects of COVID-19 on cognition and brain health. The authors highlight that COVID-19 is associated with a wide range of acute and chronic neurological, cognitive, and mental health symptoms that can persist for many months [6]. Cognitive deficits have been found in domains such as attention, executive function, and memory, even in mild cases without ongoing symptoms [10,11]. The severity of these deficits is often related to the acute severity of COVID-19 [12,13]. Brain imaging abnormalities have been observed in some patients, including reduced cortical thickness, white matter changes, and hypometabolism in frontoparietal regions [14,15,16]. These abnormalities sometimes correlate with cognitive deficits [17,18]. The authors discuss possible mechanisms for the cognitive effects, including cerebrovascular factors, dysregulated autoimmunity and neuroinflammation, and psychological impacts [19,20]. Direct viral invasion of the brain is considered unlikely [21].

Risk factors for chronic cognitive impairment include older age, severity of acute illness, and presence of ongoing symptoms [22,23]. Vaccination may reduce the risk of long-term cognitive symptoms [24,25]. The authors emphasize that there are still many unanswered questions about the trajectory of cognitive recovery, subtypes of post-COVID cognitive problems, and the evolution of brain imaging findings over time. They call for further interdisciplinary research to optimize treatment of the long-term effects on cognition and brain health.

Table 1 presents an overview of the key findings from studies investigating the impact of COVID-19 on cognitive function and brain health. The table is divided into sections covering cognitive function in the acute and chronic phases, specific cognitive domains (vigilance, executive functions, and episodic memory), the association between symptoms and objective cognitive impairments, mental health and its association with cognition, neuroimaging findings from MRI and FDG-PET, and possible underlying mechanisms. Each finding is supported by relevant references from the literature. The table highlights the persistence of cognitive deficits in some individuals, the potential impact of ongoing symptoms, and the need for further research to establish the prevalence and mechanisms of COVID-19-related brain changes. Understanding these factors is crucial for designing future studies and developing targeted interventions for those affected by the long-term cognitive and neurological consequences of COVID-19.

## 3. Brain Laboratory Biomarkers in Long COVID

Emerging evidence suggests that various biological biomarkers may be altered in patients with long COVID, potentially contributing to the neurological and cognitive symptoms observed in this condition. Several studies have investigated biomarkers such as tau, amyloid, light chains, lipids, proteins, and cytokines in the context of long COVID.

Tau and amyloid: A study by Frontera et al. [69] found that hospitalized COVID-19 patients had elevated levels of total tau, phosphorylated tau-181, and GFAP in their cerebrospinal fluid (CSF) compared to controls, suggesting potential neuronal injury and astrocytic activation. However, the study did not specifically focus on long COVID patients. Another study by Peluso et al. [70] reported that plasma neurofilament light chain (NfL) levels were elevated in COVID-19 survivors at a median of 4.5 months post-infection, correlating with cognitive impairment. The authors did not find significant differences in amyloid or tau levels between COVID-19 survivors and controls.

Light chains: A study by Verde et al. [71] found elevated kappa free light chain (KFLC) levels in the CSF of COVID-19 patients with neurological complications, suggesting intrathecal immunoglobulin synthesis and potential autoimmune processes. However, the study did not specifically focus on long COVID patients.

Lipids: A study by Fernández-Castañeda et al. [20] reported alterations in lipid metabolism in the brains of mice infected with SARS-CoV-2, including increased levels of ceramides and sphingolipids. The authors suggest that these changes may contribute to the neurological and cognitive symptoms observed in long COVID.

Proteins and cytokines: A study by Phetsouphanh et al. [72] found persistent elevation of pro-inflammatory cytokines, including IFN-β, IFN-γ, CXCL9, CXCL10, and TNF, in long COVID patients compared to controls. The authors also reported increased levels of brain-derived neurotrophic factor (BDNF) and a decrease in the neuroprotective factor NCAM1 in long COVID patients, potentially linking inflammation to neurological symptoms.

While these studies provide preliminary evidence for the involvement of various biological biomarkers in long COVID, more research is needed to establish their role in the pathogenesis of neurological and cognitive symptoms. Future studies should focus on larger cohorts of long COVID patients, employ standardized definitions and assessments, and investigate the longitudinal dynamics of these biomarkers in relation to clinical outcomes.

## 4. The Importance of Considering Long COVID in Brain Health Research

The impact of long COVID on brain health has significant implications for various research studies investigating neurological conditions, cognitive function, and brain health. In the context of brain stimulation therapies, such as transcranial magnetic stimulation (TMS) or transcranial direct current stimulation (tDCS), failing to screen for long COVID may lead to confounding effects. The neurological and cognitive symptoms associated with long COVID, such as memory deficits, attention difficulties, and executive dysfunction [73], could influence the outcomes of these stimulation interventions when used to treat cognitive impairments or enhance cognitive function. Additionally, the neuroinflammatory and neurophysiological changes associated with long COVID [3], such as persistent microglial activation or altered neural connectivity, may interact with brain stimulation techniques in unexpected ways. These interactions could potentially affect the safety and efficacy of brain stimulation treatments for various neurological disorders in individuals with a history of long COVID.

Similarly, in neuroimaging studies aimed at investigating brain structure, function, and connectivity, neglecting to consider the presence of long COVID could lead to misinterpretation of results. Neuroimaging techniques, such as magnetic resonance imaging (MRI) or positron emission tomography (PET), may reveal brain changes in individuals with long COVID that overlap with those observed in other neurological conditions [52]. For example, gray matter atrophy, white matter abnormalities, or altered brain metabolism in regions typically affected by certain disorders may also be present in individuals with long COVID, potentially leading to diagnostic confusion if long COVID status is not considered [74].

Furthermore, cognitive assessments and behavioral studies may also be impacted by the presence of long COVID symptoms. Cognitive deficits and neuropsychiatric symptoms associated with long COVID, such as attention lapses, memory problems, and mood disturbances, can influence performance on cognitive tests and other behavioral measures [51]. Failing to account for these symptoms may lead to inaccurate conclusions about the cognitive status or behavioral phenotypes of individuals with neurological conditions or other brain health concerns.

Therefore, it is crucial for researchers and clinicians to incorporate screening for long COVID history and symptoms when conducting studies related to brain health, cognitive function, and neurological disorders. This will help to ensure accurate interpretation of results, minimize confounding effects, and optimize treatment outcomes for individuals with a history of long COVID.

Despite the growing evidence of long COVID’s impact on brain health, many studies have not adapted their designs or screening processes to account for this factor. A review by Premraj et al. [75] found that most studies investigating the neurological and neuropsychiatric consequences of COVID-19 focused on the acute phase of the illness, with limited attention given to the long-term effects of the virus on brain health [75].

To mitigate these concerns and advance our understanding of long COVID’s impact on brain health, collaborative research efforts across neuroscience, psychology, psychiatry, and other relevant fields are essential. Establishing multicenter research consortia, such as the COVID-19 Neurology International Consortium (CONIC), can facilitate data sharing, standardize research protocols, and promote cross-disciplinary collaboration. Leveraging existing research networks and infrastructures, such as the Alzheimer’s Disease Research Centers (ADRCs) or the NeuroNEXT Network, can also efficiently accelerate the study of the long-term neurological and psychiatric consequences of COVID-19. Furthermore, international collaboration and data harmonization efforts are crucial, given the global nature of the COVID-19 pandemic and the potential for regional differences in Long COVID prevalence and manifestations.

## 5. The Need for Adapted Study Designs

To ensure the accuracy and reliability of brain health research findings, it is crucial for researchers to adapt their study designs to account for the potential confounding effects of COVID-19 infection and long COVID symptoms. This may involve:

1. Screening participants for a history of COVID-19 infection—researchers should include questions about prior COVID-19 infection in their screening processes, as a history of infection may influence brain health outcomes [76];
2. Assessing the presence of long COVID symptoms—participants should be screened for the presence of persistent symptoms, such as cognitive impairment, fatigue, and mood disturbances, which may be indicative of long COVID [4];
3. Stratifying analyses based on COVID-19 status—researchers should consider stratifying their analyses based on participants’ COVID-19 status to evaluate the potential impact of infection and long COVID on study outcomes [6];
4. Longitudinal assessments—incorporating longitudinal assessments into study designs can help researchers track the long-term effects of COVID-19 on brain health and identify potential interventions [10].

The challenge of identifying long COVID cases is further complicated by the fact that some individuals may experience asymptomatic infections [77]. These individuals may not be aware of their COVID-19 history, making it difficult for researchers to accurately screen for potential confounding effects. This underscores the importance of developing comprehensive screening methods and utilizing objective biomarkers, such as antibody tests, to identify individuals with a history of COVID-19 infection.

## 6. The Importance of Screening for Long COVID

Screening for long COVID symptoms is particularly important in brain health research, as the condition has been associated with a range of neurological and neuropsychiatric manifestations [78]. These may include:

1. Cognitive impairment—long COVID patients have reported persistent difficulties with memory, attention, and executive function [73];
2. Fatigue—chronic fatigue is a common symptom of long COVID and may impact cognitive performance and overall brain health [79];
3. Mood disturbances—long COVID has been associated with increased rates of depression, anxiety, and post-traumatic stress disorder (PTSD) [80].

By screening for these symptoms, researchers can better understand the potential confounding effects of long COVID on brain health outcomes and develop targeted interventions to support affected individuals. In brain stimulation studies, screening for long COVID can help researchers tailor interventions to address the specific neurological and cognitive symptoms experienced by individuals with the condition [73]. In neuroimaging studies, screening for long COVID can improve the accuracy of findings by ensuring that observed differences in brain structure or function are not confounded by the presence of persistent post-COVID symptoms [10].

To address this issue, researchers must prioritize the inclusion of long COVID screening in their study designs. This can involve incorporating questions about prior COVID-19 infection and persistent symptoms into screening processes, stratifying analyses based on participants’ COVID-19 status, and conducting longitudinal assessments to track the long-term effects of the virus on brain health [4].

## 7. Collaborative Research Efforts and Data Harmonization

To mitigate these concerns and advance our understanding of long COVID’s impact on brain health, collaborative research efforts across neuroscience, psychology, psychiatry, and other relevant fields are essential.

The COVID-19 Neurological and Psychiatric Sequelae (CNS) consortium is a global collaboration of researchers and clinicians investigating the long-term neurological and psychiatric consequences of COVID-19 [78]. The consortium aims to harmonize data collection and analysis across multiple study sites to identify risk factors, biomarkers, and potential interventions for long COVID-related neurological and psychiatric symptoms. The CNS consortium has established a standardized protocol for data collection, including detailed neurological and psychiatric assessments, cognitive testing, and biospecimen collection [78]. Preliminary findings from the consortium suggest that a significant proportion of individuals with long COVID experience persistent cognitive deficits, particularly in the domains of attention, memory, and executive function [81].

The ALBA COVID study is an international longitudinal cohort study investigating the long-term impact of COVID-19 on brain health and cognitive function [10]. The study leverages online cognitive assessments and questionnaires to collect data from participants worldwide, allowing for the identification of factors associated with cognitive impairment in long COVID and the monitoring of cognitive trajectories over time. Initial results from the ALBA COVID study indicate that individuals with long COVID perform worse on cognitive tests compared to matched controls, with deficits observed in multiple domains, including reasoning, problem-solving, and spatial working memory [6]. The study also found that the severity of acute COVID-19 illness is associated with the degree of cognitive impairment in long COVID [23].

The U.S. National Institutes of Health (NIH) launched the Post-Acute Sequelae of SARS-CoV-2 infection (PASC) initiative to investigate the long-term consequences of COVID-19 [82]. The initiative supports multiple studies investigating the biological mechanisms, risk factors, and potential interventions for long COVID. One of the PASC initiative’s key priorities is to develop a standardized set of common data elements (CDEs) for long COVID research, which will facilitate data sharing and collaboration across research teams [82]. The CDEs cover various domains, including demographics, medical history, symptoms, and functional outcomes, and are designed to ensure consistency and comparability of data across studies [83].

The potential impact of COVID-19 vaccinations on long COVID is an important consideration in brain health research. Emerging evidence suggests that vaccination may reduce the risk and severity of long COVID. A study by Antonelli et al. [24] found that individuals who received two doses of COVID-19 vaccine had a lower risk of developing long COVID compared to unvaccinated individuals [24]. Furthermore, vaccinated individuals who did develop long COVID reported less severe symptoms and a shorter duration of illness compared to their unvaccinated counterparts [24]. These findings highlight the potential protective effect of vaccination against long COVID, although more research is needed to fully understand the impact of vaccines on long-term neurological and psychiatric outcomes.

## 8. Conclusions and Future Directions

In conclusion, considering long COVID in brain health and dementia research, particularly in brain stimulation and neuroimaging studies, is essential for ensuring the accuracy and reliability of findings, developing targeted interventions for individuals with persistent symptoms, and contributing to a better understanding of the long-term effects of COVID-19 on brain health. As the prevalence of long COVID continues to grow, it is crucial for researchers to adapt their methods to account for this important factor in their work.

**Figure 1:**
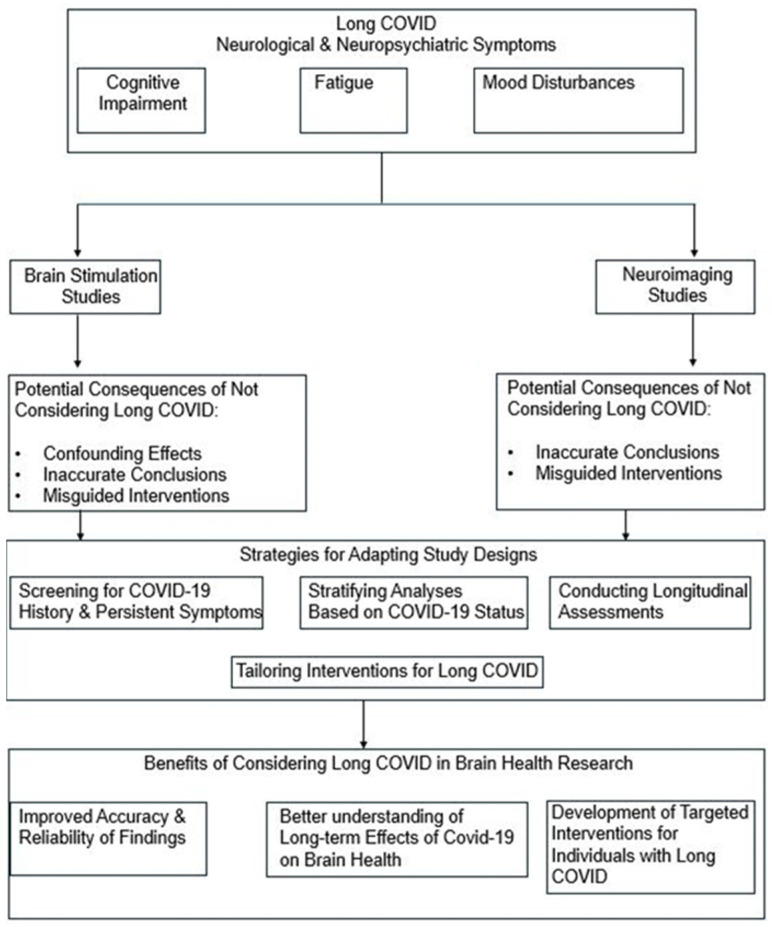
The Impact of Long COVID on Brain Health Research: A Roadmap for Adaptation.

The diagram presented in this manuscript (Figure 1) provides a comprehensive overview of the key considerations and action steps needed to address the challenge of long COVID in brain health research, with a specific focus on brain stimulation and neuroimaging studies. For example, in the context of brain stimulation therapies, such as transcranial magnetic stimulation (TMS) or transcranial direct current stimulation (tDCS), the diagram highlights the importance of screening for long COVID symptoms to ensure that the presence of persistent neurological or psychiatric symptoms does not confound treatment outcomes [84]. Similarly, in neuroimaging studies aimed at investigating brain structure and function in the context of long COVID, the diagram emphasizes the need to adapt study designs to account for the potential impact of persistent symptoms on brain health outcomes [14].

Moving forward, several key priorities should guide future research on long COVID and brain health, as outlined in the diagram:

1. Develop and validate standardized screening tools and diagnostic criteria for long COVID, considering the wide range of neurological and psychiatric symptoms associated with the condition. This will enable more accurate identification of individuals with long COVID and facilitate comparisons across studies [85].
2. Investigate the mechanisms underlying the neurological and psychiatric manifestations of long COVID, including the role of inflammation, autoimmunity, and direct viral effects on the brain. Understanding these mechanisms will inform the development of targeted therapies and interventions, such as novel brain stimulation protocols or neuroimaging-guided treatments [86].
3. Identify risk factors and protective factors for long COVID, including demographic, clinical, and genetic factors. This knowledge will help stratify patients based on their risk profiles and guide personalized prevention and treatment strategies, which may involve tailored brain stimulation interventions or neuroimaging-based monitoring approaches [5].
4. Conduct longitudinal studies to track the long-term trajectory of neurological and psychiatric symptoms in individuals with long COVID, as well as the impact of these symptoms on cognitive function, quality of life, and overall brain health. Such studies, which may incorporate serial brain stimulation assessments or neuroimaging evaluations, will provide valuable insights into the natural history of long COVID and inform the timing and duration of interventions [51].
5. Develop and test targeted interventions for the neurological and psychiatric symptoms of long COVID, including cognitive rehabilitation, symptom-specific medications, and lifestyle modifications. Rigorous clinical trials are needed to establish the safety and efficacy of these interventions, which may be combined with brain stimulation therapies or guided by neuroimaging findings, to optimize treatment protocols [87].
6. Foster international collaboration and data sharing through the establishment of research consortia, standardized protocols, and open-access data platforms. Such efforts, which should include harmonized brain stimulation and neuroimaging methodologies, will accelerate the pace of discovery and ensure that findings are globally applicable and generalizable [88].

Addressing these priorities, as illustrated in the diagram, will require a concerted effort from the neuroscience community, policymakers, and funding agencies. By working together to advance our understanding of long COVID and its impact on brain health, particularly through the lens of brain stimulation and neuroimaging studies, we can develop evidence-based strategies to support the millions of individuals affected by this condition and mitigate its long-term consequences.

## Figures and Tables

**Table 1:** Summary of the main findings on the effects of COVID-19 on cognition and brain health.

Topic	Main Findings
Acute phase cognition (<3 months)	- Impairments found using cognitive screening tests, especially in those over 59 years old and hospitalized patients [26,27]; - Deficits scaled with acute COVID-19 severity [10]; - Most affected domains were semantic reasoning and multi-stage planning [10]; - Executive dysfunction and memory deficits commonly reported [18,27,28,29,30,31,32,33].
Chronic phase cognition (>3 months)	- Attention, executive function, and memory deficits can persist, potentially up to 2 years in those with ongoing symptoms [11,14,27,29,34,35,36,37,38,39,40,41,42,43]; - Prevalence is unclear, but cognitive impairment more common in multiple domains vs. single domain [27,35,44]; - Majority of mild-to-moderate cases recover within first year [22,45,46,47]; - Presence of ongoing symptoms a key factor in persistence of deficits [23,34,40].
Vigilance and sustained attention	- Significant impairment in detecting targets on simple tasks, even in young individuals without subjective complaints [11,38].
Executive functions	- Trail-making task impairment at 7 months in severe cases, and worse with greater age in milder cases [11,12,13,37]; - Younger people showed no chronic effects on trail-making tasks or other executive functions [11,37,40,45,48].
Episodic memory	- Deficits irrespective of stimulus type (words or pictures), even in young mild cases without ongoing symptoms [11,27,29,39,40,41,49,50].
Relation of symptoms to objective cognitive impairments	- Ongoing cognitive symptoms a strong predictor of objective deficits [23,34,40,43]; - But deficits can occur in absence of symptoms, and association not always found [9,36].
Mental health and cognition	- COVID-19 is linked to high rates of sleep disturbance, anxiety, and mood disorders which impact cognition [10,13,23,45]; - But severity of mental health symptoms not consistently correlated with degree of cognitive deficit [10,11,23,36,37,44,50].
Dementia risk	- Long COVID may contribute to the development of dementia [51,52].
MRI (Acute phase)	- White matter abnormalities frequently reported, potentially due to micro/macrovascular insults, infection, inflammation, or autoimmunity [53,54,55,56]; - But the majority had normal MRI even with acute neurological symptoms [57]; - One study found reduced intra-/extra-axonal volumes and increased free water (vasogenic edema) in frontoparietal regions [16]; - Another found thalamic susceptibility and increased mean diffusivity in thalamic radiation and sagittal stratum at 2–3 months [18].
MRI (Chronic phase)	- UK Biobank study found reduced cortical thickness in areas functionally connected to olfactory cortex and increased diffusivity in orbitofrontal cortex, anterior cingulate, insula, and amygdala at 5 months [14]; - At 1 year, one study found diffusion abnormalities in corpus callosum, corona radiata, and superior longitudinal fasciculus, especially in ICU patients [7]; - Another at 1 year found reduced hippocampal volumes (greatest in hospitalized patients) associated with impaired cognitive performance [58].
FDG-PET	- Frontoparietal hypometabolism consistently reported in patients with acute neurological symptoms [15,59,60,61]; - Longitudinal studies show this tends to resolve by ~6 months [17,48,60,61]; - Other affected areas include thalamus, insula, medial temporal lobe [62,63,64]; - Cerebellum, pons, brainstem show mixed hyper- and hypometabolism [15,17,59,60,63,64]; - Pattern of frontoparietal hypometabolism aligns with impairments in attention and executive function [65,66].
Imaging and cognitive impairment	- Found across modalities, including task-based fMRI, resting-state fMRI, gray matter volume, white matter integrity [14,15,16,27,58,67,68]; - One study found PCC patients had overall greater activation on n-back task, correlating with symptom severity [58]; - Another found reduced connectivity between parahippocampal regions and orbitofrontal-cerebellar connectivity at 11 months, with orbitofrontal changes relating to memory [67].
